# Long but Unreal Lockdowns in Latin America. Comment on Chen, Y.T.; Yen, Y.F.; Yu, S.H.; Su, E.C. An Examination on the Transmission of COVID-19 and the Effect of Response Strategies: A Comparative Analysis. *Int. J. Environ. Res. Public Health* 2020, *17*, E5687

**DOI:** 10.3390/ijerph17218064

**Published:** 2020-11-02

**Authors:** Alvaro J. Idrovo

**Affiliations:** Public Health Department, School of Medicine, Universidad Industrial de Santander, Carrera 32 #29-31, Bucaramanga 680002, Santander, Colombia; idrovoaj@yahoo.com.mx

**Keywords:** coronavirus, pandemic, lockdown, Latin America, democracy

## Abstract

Lockdowns have been important elements of epidemic control over time. During the COVID-19 pandemic, they have been implemented in many countries, at very different times, and accompanied by school or workplace closures, restrictions on mass gatherings, and public transport closure in different combinations. Recent evidence published in the *International Journal of Environmental Research and Public Health* suggests that SARS-CoV-19 transmission is diminished when strict lockdowns, contact tracing, and good public cooperation are implemented. However, in Latin America, not all lockdowns are real, and rapid increases in a few weeks in the number of infected, hospitalized, and deceased populations have been observed. In these cases, the effect of lockdowns is weakening of democracy.

Lockdowns have been important elements of epidemic control over time [1]. During the COVID-19 pandemic, they have been implemented in many countries, at very different times, and accompanied by school or workplace closures, restrictions on mass gatherings, and public transport closure in different combinations [2]. Recent evidence published in the *International Journal of Environmental Research and Public Health* suggests that SARS-CoV-19 transmission is diminished when strict lockdowns, contact tracing, and good public cooperation are implemented [3].

In Latin America, lockdowns were one of the most used strategies to have time to improve response capacity. Most countries decreed lockdowns in the second half of March, which in some countries has lasted for more than three months; in this way, they constitute the longest lockdowns together with India. According to the Government Response Stringency Index by the Oxford COVID-19 Government Response Tracker [4], countries such as Argentina, Bolivia, Venezuela, Peru, Ecuador, Colombia, and Paraguay had a very strict response to COVID-19 pandemic, including lockdowns.

However, in general, the required preparation was not achieved. Improvements in public health surveillance, such as increasing the number of qualified field epidemiologists, increasing the capacity of laboratories to meet the growing demands for diagnostics, the implementation of technologies to facilitate surveillance, and community actions to communicate and educate various communities in health were not achieved; nor was it possible to increase the number of hospital beds and intensive care units in the dimension that was initially calculated [5]. Additionally, the difficult social conditions in Latin America, which include low education levels and high levels of poverty, unemployment, and informality, led to a double discourse—on the one hand, maintaining the lockdown as a measure to prevent transmission of the infection, but at the same time, making the lockdown more flexible to reactivate the weak economy.

The effects have been somewhat evident, with rapid increases in a few weeks in the number of infected, hospitalized, and deceased populations. For this contradiction, in Latin America, not all lockdowns are real, and many news in newspapers and magazines have evidenced the breach of lockdowns (for instance, in *The Telegraph*, “lockdowns on the continent have been some of the longest on the planet, but even as infections and deaths continue to rise, quarantine measures are now being relaxed across the continent as the lives versus livelihoods debate lingers on” [6]. Evidence is clear in showing that the successful use of lockdowns is not determined by its political declaration, but by its slow, flexible, and intelligent completion. In some Latin American countries, exaggerated prolongation has potential negative political repercussions on democracy, without showing great positive effects on the control of the pandemic. The beneficial effects of democracy on population health are well known [7].
